# Crystallographic and physicochemical characterization of salcaprozoic acid: a structural basis for SNAC-enabled drug delivery systems

**DOI:** 10.1107/S2053229625008691

**Published:** 2025-10-06

**Authors:** Parag Roy, Paul G. Waddell, Rajdeep Dey, Oisín N. Kavanagh

**Affiliations:** a School of Pharmacy Newcastle University Newcastle upon Tyne NE1 7RU United Kingdom; b School of Natural and Environmental Sciences, Newcastle University, Newcastle upon Tyne, NE1 7RU, United Kingdom; c Department of Pharmaceutical Chemistry Institute of Pharmacy Nirma University Ahmedabad 382 481 India; University of Strathclyde, United Kingdom

**Keywords:** salcaprozoic acid, salcaprozate sodium, SNAC, HNAC, crystal structure, drug delivery system, oral permeation enhancer

## Abstract

Salcaprozate sodium (SNAC) is a clinically approved oral permeation enhancer, notably used in the formulation of oral semaglutide. Despite its pharmaceutical importance, the crystallographic information of SNAC or its free acid form, salcaprozoic acid {systematic name: 8-[(2-hy­droxy­phen­yl)formamido]­octa­noic acid, C_15_H_21_NO_4_, denoted HNAC}, has not been reported previously. Here, we present the first crystallographic and physicochemical characterization of HNAC using single-crystal X-ray diffraction and com­plementary analytical techniques. The structure reveals the mol­ecular conformation, hy­dro­gen-bonding network and packing features of HNAC, supported by a com­plementary solid-state dataset. These findings provide fundamental insights into the structural and physicochemical properties of this physiologically relevant form of SNAC.

## Abstract 2

This study presents the first com­prehensive crystallographic characterization of salcaprozoic acid (HNAC), the free acid form of salcaprozate sodium (SNAC), using single-crystal X-ray diffraction. A well-organized hy­dro­gen-bonding framework underpins the robust crystal packing and thermal stability of the com­pound. Complementary IR spectroscopy highlighted the impact of hy­dro­gen bonding on vibrational modes.

## Introduction

Salcaprozate sodium (Na^+^·C_15_H_20_NO_4_^−^, SNAC) is a synthetic derivative of salicylic acid, known as sodium 8-[(2-hy­droxy­benzo­yl)amino]­caprylate. It has drawn significant attention as a permeation enhancer to facilitate the oral administration of therapeutics, particularly for macromolecules that typically exhibit poor gastrointestinal absorption (Twarog *et al.*, 2019 ▸). The amphiphilic structure of SNAC facilitates transcellular drug transport by modulating the fluidity of the epithelial membrane and pH microenvironment at the site of absorption, thereby enhancing absorption of co-administered mol­ecules without causing long-term mucosal damage (Kom­mineni *et al.*, 2023 ▸). SNAC has been used in formulations of drugs that have undergone clinical trials and has achieved Generally Recognized As Safe (GRAS) status, with US Food and Drug Administration (FDA) approval for use in medical and food products (Castelli *et al.*, 2011 ▸). The clinical significance of SNAC is exemplified by its incorporation into the oral for­mulation of semaglutide, a glucagon-like peptide-1 (GLP-1) receptor agonist (Solis-Herrera *et al.*, 2024 ▸). The key part of the mechanism of action of SNAC involves neutralizing the local pH in the stomach and initiating monomerization of the peptide, thereby stabilizing semaglutide and reducing its degradation. Under physiological conditions, SNAC could dis­sociate into its free acid form, salcaprozoic acid (C_15_H_21_NO_4_, HNAC) and sodium ion com­ponents (Rebollo *et al.*, 2025 ▸).

Despite its extensive application in drug delivery systems, to the best of our knowledge, no reports of com­prehensive crystallographic studies of SNAC or its free acid form HNAC, have been reported to date. Knowledge of the crystallographic information could be useful as the structural properties are directly relevant to its *in vivo* behaviour (Datta & Grant, 2004 ▸). A deeper understanding of the mol­ecular conformation and inter­actions in the solid state can provide insights into the intrinsic physicochemical characteristics which could be bene­ficial for pharmaceutical development and mechanistic modelling of structurally related permeation enhancers.

In this study, we report the single-crystal X-ray structure of HNAC (Scheme 1), revealing its mol­ecular conformation, hy­dro­gen-bonding network and crystal packing features, along with other solid-state characterization studies. This represents the first crystallographic characterization of the free acid form and offers a structural basis for future investigations into medium-chain fatty-acid-related mol­ecular systems. During our work, we also synthesized SNAC; however, despite multiple crystallization attempts, we were unable to obtain single crystals suitable for X-ray diffraction. This further underscores the significance of the present result, as it provides valuable structural insight into a key physiologically relevant form of this important permeation enhancer.
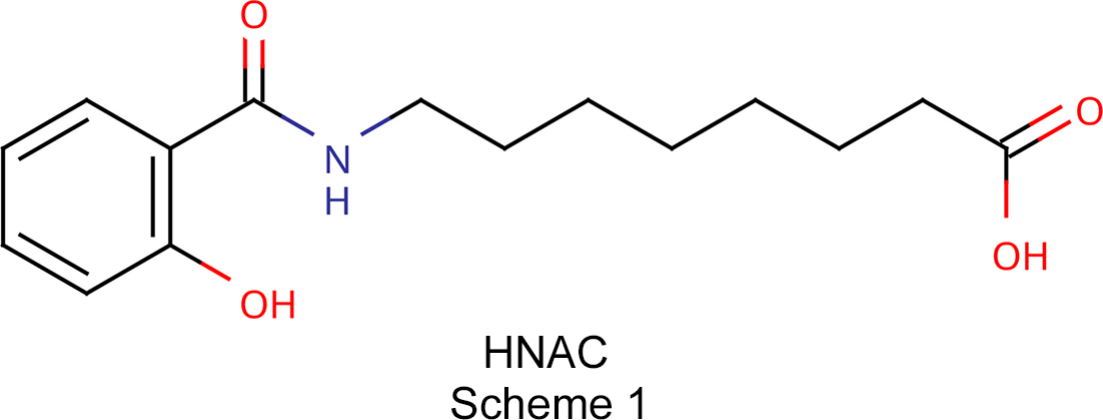


## Experimental

### Single-crystal X-ray diffraction (SCXRD) data collection and refinement

Details of the crystal structure refinement and refinement statistics for HNAC are given in Table 1 ▸. The sample of HNAC (≥98% purity) was procured from Synlyfe Research Lab­or­a­tory (Gujarat, India). Crystals of HNAC were obtained by slow evaporation of a methano­lic solution at room tem­per­a­ture (≃ 298 K). A 5 ml sample of the com­pound dissolved in methanol was left undisturbed, and solvent evaporation over a period of 5–6 d yielded crystals suitable for analysis.

H atoms were positioned with idealized geometry, with the exception of those bound to heteroatoms, the positions of which were located using peaks in the Fourier difference map, with their coordinates allowed to refine freely. The displacement parameters of the H atoms, *U*_iso_, were constrained using a riding model, with *U*_iso_(H) set to be an appropriate multiple of the *U*_eq_ value of the parent atom.

### Powder X-ray diffraction

Powder X-ray diffraction patterns were recorded using an Empyrean diffractometer (Malvern Panalytical, UK) in Bragg–Brentano θ/θ geometry, equipped with a PIXcel 3D detector and a reflection–transmission spinner stage. The instrument utilized monochromatic Cu *K*α_1_ radiation (λ = 1.54184 Å) generated at 40 kV and 40 mA, with a take-off angle of 6.0°. Data were collected at room tem­per­a­ture over a 2θ range of 5 to 40°, with a step size of 0.01° and 20 s per step. The obtained diffractograms were analyzed using *HighScore Plus* (Malvern Panalytical, UK) software (Degen *et al.*, 2014 ▸). Rietveld refinement was performed using the structural model derived from single-crystal data, allowing accurate determination of lattice parameters, phase identification and confirmation of the sample purity.

### Thermal analysis

Differential scanning calorimetry (DSC) was carried out using a TA Instruments Q2000 system (Cheshire, UK) over a tem­per­a­ture range of 30–300 °C. Approximately 2–5 mg of each sample were weighed into hermetically sealed aluminium pans and heated at a rate of 10 °C min^−1^ under a constant nitro­gen purge (50 ml min^−1^) to prevent oxidative degradation. The resulting thermograms were analysed using TA Instruments *Universal Analysis* software (https://www.tainstruments.com/).

Thermogravimetric analysis (TGA) was also performed to assess the decom­position behaviour of the samples. Mea­surements were conducted under a nitro­gen atmosphere using a TA Instruments Q500 system (Cheshire, UK). 2–5 mg of samples were weighed into aluminium pans and heated from 30 to 500 °C at a constant heating rate of 10 °C min^−1^. The resulting thermograms were analysed using TA Instruments *Universal Analysis* software.

### Fourier–transform IR spectroscopy

IR spectra were recorded using an Agilent Cary 630 FT–IR spectrometer equipped with a diamond attenuated total re­flectance (ATR) accessory. The instrument was operated with *MicroLab* FT–IR software (https://www.agilent.com/en/product/mol­ecular-spectroscopy/ftir-spectroscopy) for spectral acquisition and processed by *OriginPro* software (Origin­Lab Corporation, 2022 ▸). Samples were analysed in their solid form without further preparation. Each spectrum was obtained over the range 4000–650 cm^−1^. The obtained spectra were pro­cessed to identify characteristic functional group vibrations.

### Synthesis of SNAC

HNAC (100 mg) was transferred to a 25 ml round-bottomed flask equipped with a magnetic stirrer bar. Iso­propanol (≥99.5% purity, 5.0 ml) was added and the mixture was heated to 50 °C with constant stirring until a clear solution was obtained. An aqueous solution of sodium hydroxide (1 *M*) was added dropwise over a period of 5 min under continuous stirring until the pH reached 9–10, at which point a white precipitate of SNAC began to form. The reaction mixture was stirred at room tem­per­a­ture for an additional 30 min, and the precipitate was collected by vacuum filtration and dried at ambient tem­per­a­ture. PXRD (Fig. S1 in the supporting information) and DSC analysis confirmed the material as polymorphic form II (Levchik *et al.*, 2016 ▸). However, despite multiple crystallization attempts from various solvent systems, we were unable to obtain single crystals suitable for X-ray diffraction analysis.

## Results and discussion

### Cystal structure analysis

**Figure 1:**
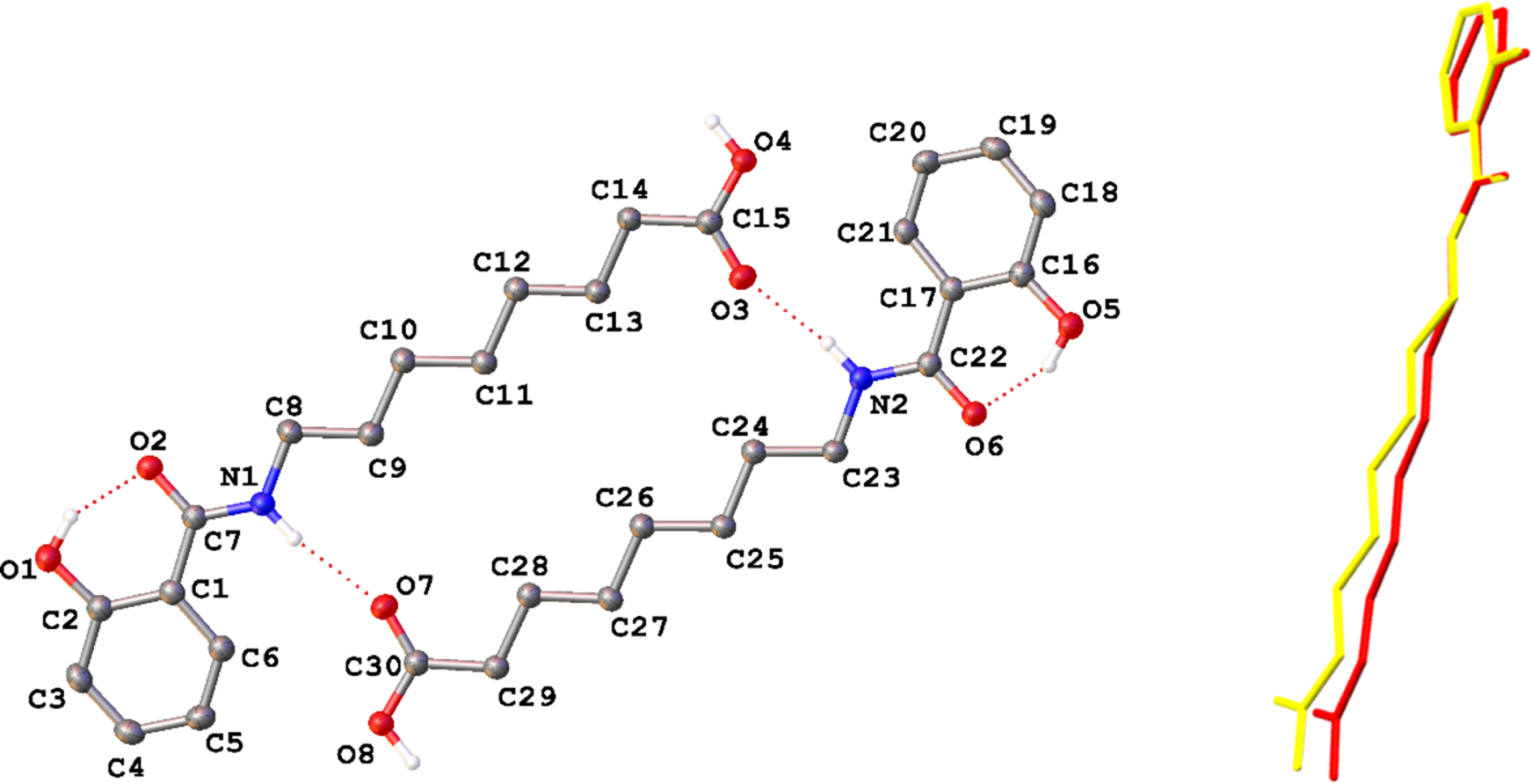
The asymmetric unit of HNAC, with selected atom labelling, showing (left) displacement ellipsoids plotted at the 50% probability level and (right) an overlay of the two symmetry-independent mol­ecules. H atoms bound to C atoms have been omitted for clarity.

HNAC crystallizes in the monoclinic space group *P*2_1_/*c* (Fig. 1 ▸), with two crystallographically independent mol­ecules in the asymmetric unit (*Z*′ = 2) that are related by approximate inversion symmetry. Overlaying the two mol­ecules further demonstrates this relationship, revealing two distinct conformations.

**Figure 2:**
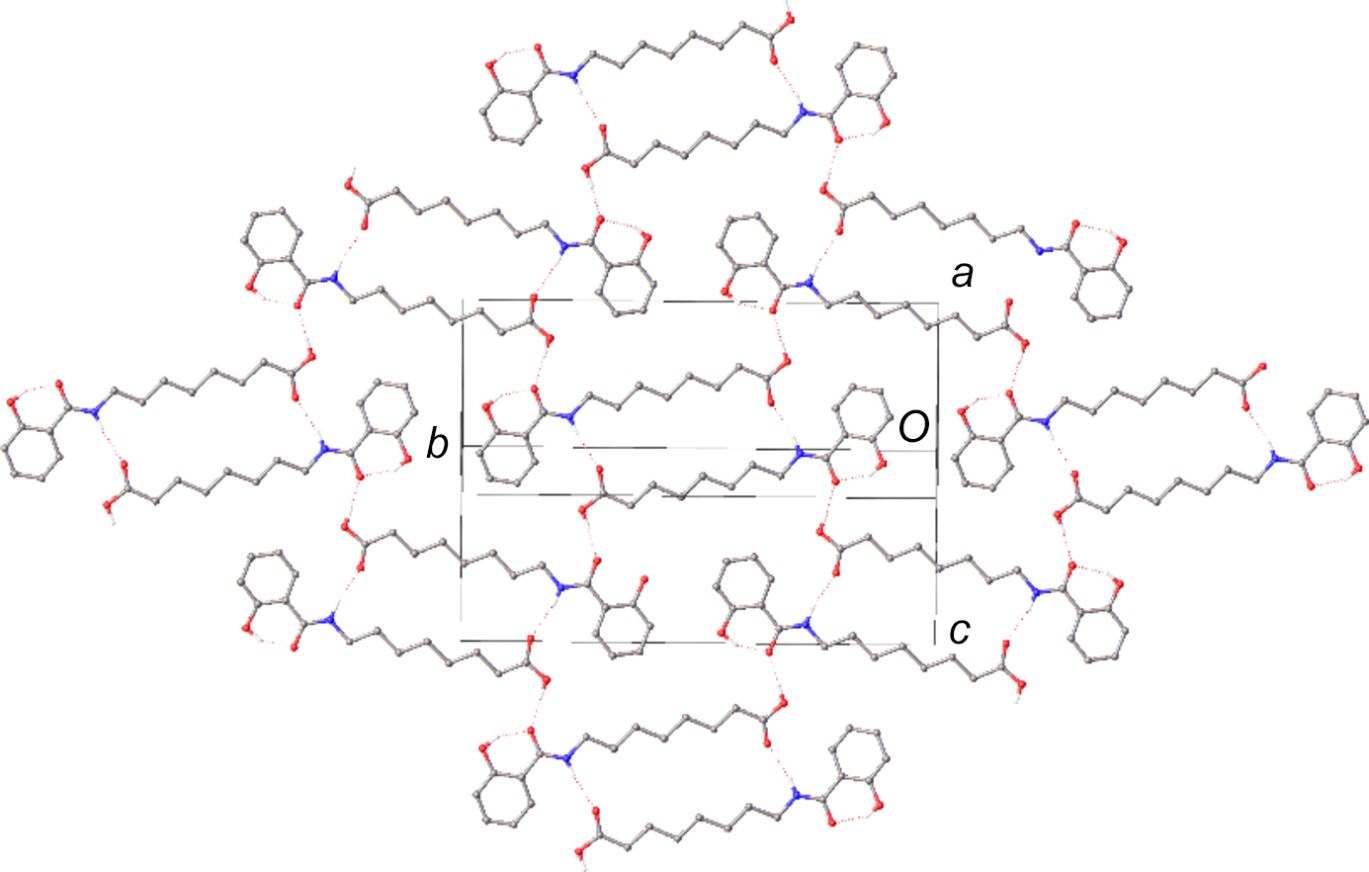
The 2D hy­dro­gen-bonded layer in the (101) plane of the crystal structure of HNAC. H atoms bound to C atoms have been omitted for clarity.

The asymmetric unit is held together by hy­dro­gen bonds (Table 2 ▸) in which the amide proton donates to the carbonyl O atom of the carb­oxy­lic acid group to form a ring motif with the graph set 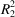
(22) (Etter, 1990 ▸). These rings are linked by further hy­dro­gen bonds, where the carb­oxy­lic acid group acts as a donor to the carbonyl O atom of the amide, forming a 2D network coplanar with the crystallographic (101) plane (Fig. 2 ▸). There do not appear to be any salient inter­molecular inter­actions between these hy­dro­gen-bonded layers.

In addition, an intra­molecular hy­dro­gen bond forms between the hydroxyl group and the amide carbonyl group, as would be anti­cipated according to Etter’s second rule of hy­dro­gen bonding (Etter, 1990 ▸).

In the absence of an experimentally resolved structure for SNAC, despite several attempts, the structure of HNAC can instead be com­pared with known examples in the Cambridge Structural Database (CSD; Groom *et al.*, 2016 ▸) that share similar fragments and functionalities. The structures of other medium-chain fatty acids which acts as permeation enhancers like hepta­noic acid (Bond, 2004 ▸; CSD refcode ISENOJ) and suberic acid (Mishra *et al.*, 2015 ▸; SUBRAC12) exhibit the same anti­periplanar arrangement along the length of the carbon chain as that observed in HNAC, though in these instances hy­dro­gen bonding is observed between carb­oxy­lic acid groups forming 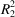
(22) ring motifs. This is likely attributable to the absence of alternative donor or acceptor sites in these structures, unlike in HNAC, where multiple options are available.

In terms of the 2-hy­droxy­benzoyl­amino moiety, few examples are available that possess a similar saturated carbon chain with terminal hy­dro­gen-bond donors or acceptors, with the closest being 2-hy­droxy-*N*-(2-hy­droxy­eth­yl)benzamide (CSD refcode family EVIWIQ). Among the two available examples of this structure in the CSD (Betz *et al.*, 2011 ▸; Wanke *et al.*, 2011 ▸), the entry EVIWIQ01 reported by Wanke and co-authors was selected for com­parison, as its data were collected at the same tem­per­a­ture as HNAC (150 K).

**Figure 3:**
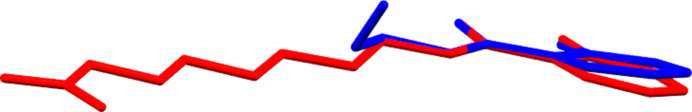
An overlay of one of the independent mol­ecules of HNAC (red) and that of EVIWIQ01 (blue). H atoms have been omitted for clarity.

Considering the structural fragment that both HNAC and EVIWIQ01 share, the two exhibit relatively similar conformations (Fig. 3 ▸). Where they do differ is in the orientation of the benzene ring relative to the amide moiety. Where in HNAC the N1—C7—C1—C6 torsion angles are 4.43 (15) and 6.45 (15)°, the equivalent torsion in EVIWIQ01 is 12.2 (2)°, representing a significant deviation from planarity.

This can be rationalized by considering the relative directions of the hy­dro­gen bonds in each structure. In HNAC, the bonds are all essentially in the plane of the hy­dro­gen-bonding network [*i.e.* the (101) plane]. However, in EVIWIQ01, though the structure ultimately forms a 2D network in the (100) plane, the hy­dro­gen bonds are not in this plane (*e.g.* O3—H4⋯O2 forms an angle of *ca* 22° with the *b* axis). These attractive forces therefore act at an angle to the plane of the mol­ecule and lead to greater distortions of the otherwise planar geometry. The absence of similar inter­actions in HNAC allows it to form the almost perfectly planar sheets observed in the structure.

### Powder X-ray diffraction and Rietveld refinement

**Figure 4:**
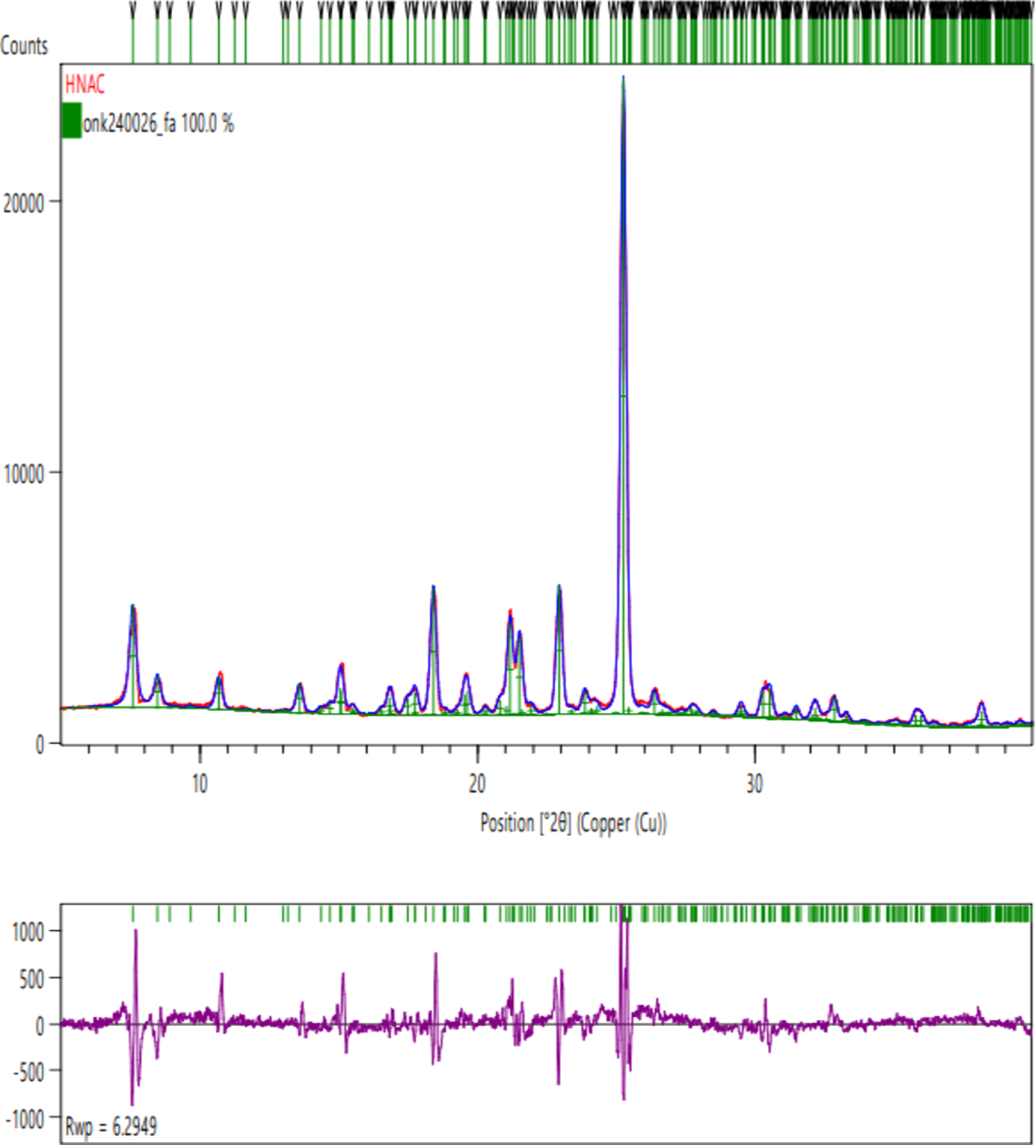
Rietveld refinement of the PXRD pattern of HNAC. Colour code: red = observed data; blue = calculated profile; purple = difference plot; green vertical tick marks = Bragg reflection positions.

The crystalline structure of HNAC was confirmed by Rietveld refinement of the PXRD data using *HighScore Plus* (Fig. 4 ▸). The experimental diffraction pattern was measured using monochromatic Cu *K*α_1_ radiation (λ = 1.54184 Å) and refinement was performed against the single-phase structural model derived from the CIF. Closer inspection of the diffraction profile revealed weak shoulder features, notably near 7.5° 2θ. These could be accounted for within the Rietveld model as overlapping reflections from the same monoclinic phase, combined with low-angle axial divergence effects. The refinement converged successfully with good agreement between the calculated and experimental patterns. The goodness-of-fit (GoF) was 2.541, with the weighted profile *R* factor (*R*_wp_) = 6.295%, profile *R* factor (*R*_p_) = 4.708% and Bragg *R* factor = 2.001%. The expected *R* factor was 2.477%, indicating a well-fitted model with no overfitting. All Bragg reflections could be indexed to a single monoclinic phase, *P*2_1_/*c*, and no additional peaks were detected. The refined unit-cell parameters from the PXRD data (*a* = 10.23430, *b* = 23.73930, *c* = 12.04533 Å and β = 100.0874°) closely match those from the SCXRD data. The Rietveld refinement confirms that the sample is crystalline and structurally consistent with the reference CIF which supports the reasoning that the shoulders arise from intrinsic profile broadening rather than from impurities or an additional polymorph.

### Thermal analysis

**Figure 5:**
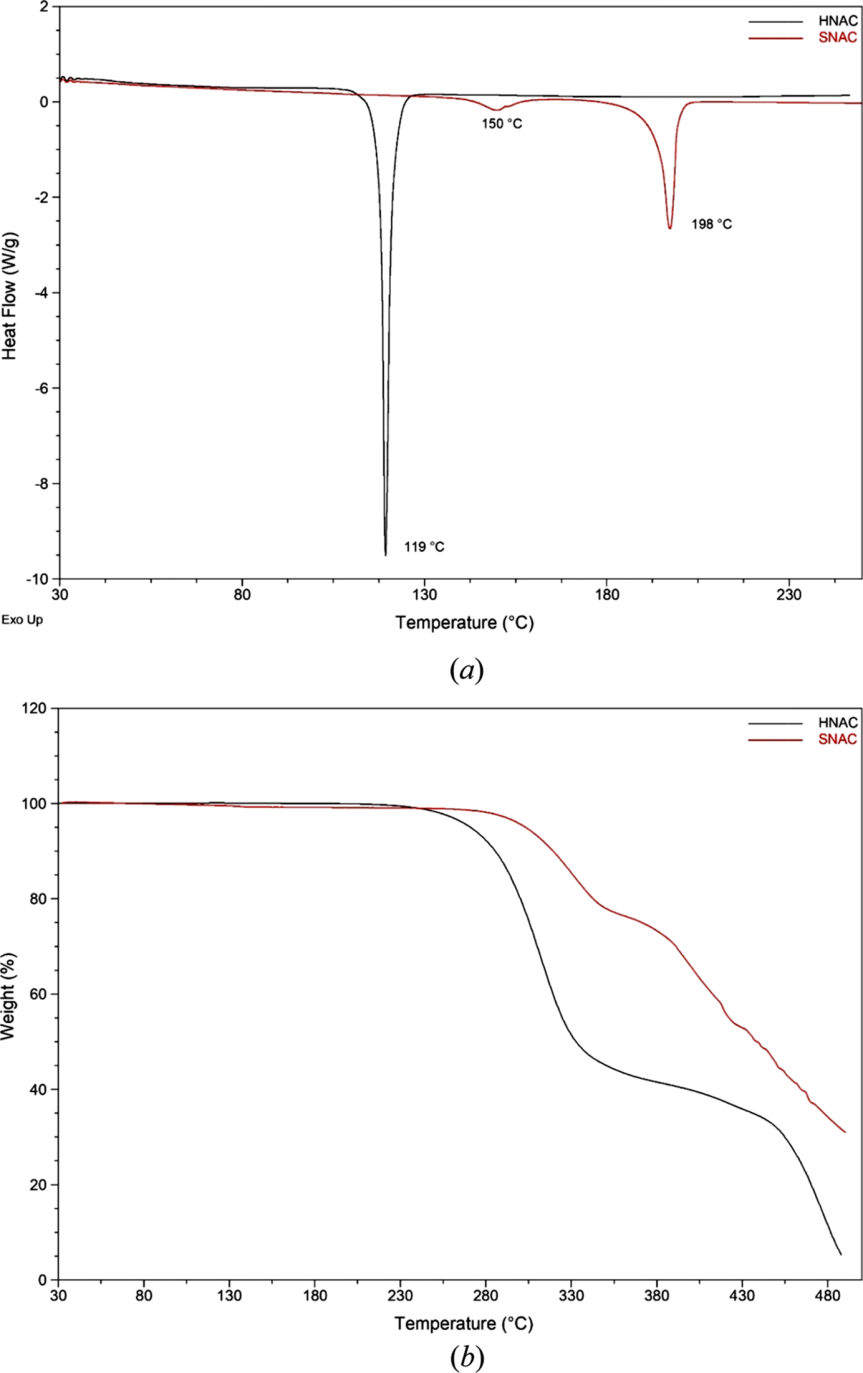
Combined (*a*) DSC thermogram and (*b*) TGA endotherm of HNAC.

The thermal behaviours of HNAC and SNAC were investigated by DSC and TGA (Fig. 5 ▸). HNAC exhibits a single sharp endotherm at approximately 119 °C, indicative of the melting point, which is consistent with the existing literature reports (Rebollo *et al.*, 2025 ▸). The TGA curve of HNAC shows a rapid and substantial weight loss immediately following melting, suggesting that decom­position is triggered directly after the phase transition. SNAC demonstrates a minor endothermic peak at approximately 150 °C and a sharp endothermic peak at approximately 198 °C, characteristic of polymorphic form II (Levchik *et al.*, 2016 ▸). The TGA profile confirms that SNAC undergoes a two-stage decom­position process, with initial mass loss beginning after 198 °C, followed by progressive degradation at higher tem­per­a­tures. The thermal profiles are consistent with the mol­ecular structures of the HNAC and SNAC. For HNAC, the neutral carb­oxy­lic acid form, melting at 119 °C, appears to assist rapid volatilization and breakdown of the mol­ecular framework, possibly through deca­rboxylation and cleavage of the amide linkage. Whereas the ionic stabilization of the carboxyl­ate group of SNAC delays the decom­position to higher tem­per­a­tures. The stepwise mass losses observed for SNAC suggest that the initial stage may involve fragmentation of more labile substituents, followed by degradation of the aliphatic chain and aromatic core.

### IR spectroscopy

The IR spectra of HNAC and SNAC provide valuable insights into the structural and electronic differences induced by deprotonation and salt formation. These changes are particularly evident in the regions corresponding to O—H, C=O, C—N and aromatic functionalities.

**Figure 6:**
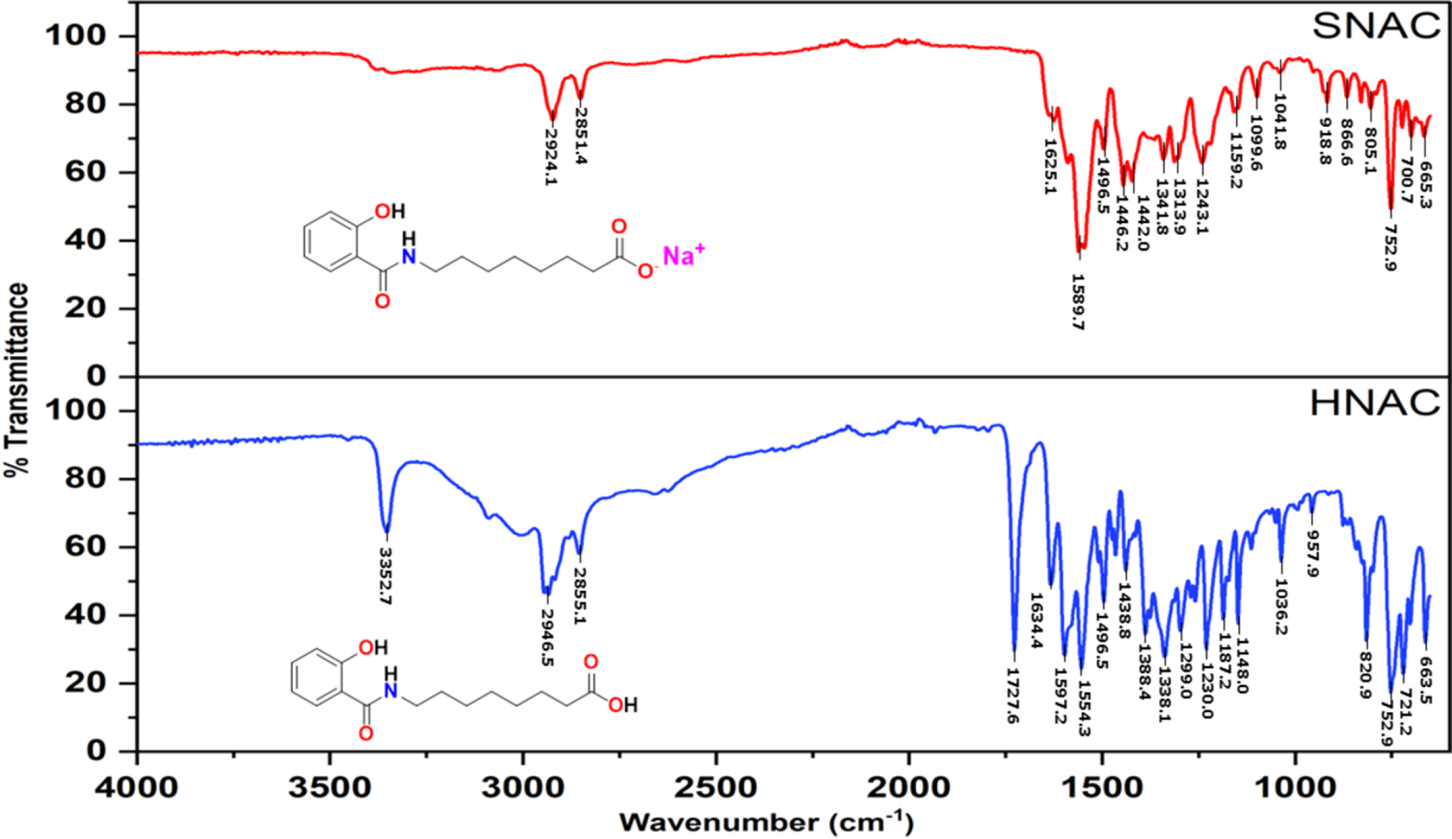
FT–IR spectra of HNAC and SNAC.

In the IR spectrum of HNAC (Fig. 6 ▸), a broad and intense band centred around 3352 cm^−1^ is attributed to the O—H stretching vibration of the carb­oxy­lic acid group (Shen *et al.*, 2024 ▸). This broadness is indicative of strong hy­dro­gen bonding, which is characteristic of carb­oxy­lic acids (Langner & Zundel, 1995 ▸). Additionally, the phenolic –OH group may also contribute to this band, but its involvement in inter­molecular hy­dro­gen bonding with neighbouring mol­ecules could influence its precise position and intensity (Yamashita & Takatsuka, 2007 ▸). Upon the formation of the sodium salt, SNAC, the O—H stretching band is significantly diminished or absent, indicating deprotonation of the carb­oxy­lic acid and the formation of the carboxyl­ate anion (COO^−^). The disappearance of this peak is consistent with ionic salt formation, where the H atom is replaced by a sodium ion (Na^+^), resulting in altered vibrational characteristics. Moreover, the phenolic –OH group may undergo extensive inter­molecular hy­dro­gen bonding, especially in the solid state, which can either shift the absorption to lower frequencies or render it IR-inactive due to reduced dipole change during vibration (Dai *et al.*, 2023 ▸).

The C=O stretching vibration is another critical diagnostic region. In the free acid, a strong sharp absorption at 1727 cm^−1^ corresponds to the carbonyl (C=O) stretch of the carb­oxy­lic acid group. This peak is notably absent or shifted in the SNAC spectrum. Instead, a prominent band appears around 1589 cm^−1^, which is characteristic of the asymmetric stretching of the carboxyl­ate anion (COO^−^) (Max & Chapados, 2004 ▸). This shift to lower wavenumbers reflects the delocalization of negative charge across the two O atoms in the carboxyl­ate group, reducing the bond order and thus lowering the stretching frequency (Dey *et al.*, 2025 ▸). Additionally, the amide functional group is evident in both spectra. The amide II band, arising from N—H bending coupled with C—N stretching, is observed in the range 1597–1554 cm^−1^. Hydrogen bonding or conjugation with the aromatic ring may influence the exact position and intensity of this band. The C—N stretching vibration of the amide is detected near 1299 cm^−1^, further confirming the presence of the amide moiety.

Aromatic features are clearly present in both spectra. The C=C stretching vibrations of the aromatic ring are observed as multiple medium-intensity bands in the range 1450–1600 cm^−1^, consistent with a substituted benzene ring system. The C—O stretching vibrations, arising from both phenolic and carb­oxy­lic acid groups, occur near 1230 cm^−1^, although these can sometimes overlap with C—N stretches depending on the local environment and substitution pattern. The aliphatic C—H stretching vibrations are represented by strong bands around 2945 and 2924 cm^−1^, common to both the acid and salt forms. These arise from the symmetric and asymmetric stretching modes of CH_2_ groups in the aliphatic chain. Furthermore, aromatic C—H out-of-plane bending vibrations appear around 870 cm^−1^, supporting the presence of mono- or disubstituted benzene rings, which are key structural motifs in HNAC and SNAC (Lin-Vien *et al.*, 1991 ▸).

## Conclusions

This study presents the first com­prehensive crystallographic characterization of salcaprozoic acid (HNAC), the free acid form of salcaprozate sodium (SNAC), using single-crystal X-ray diffraction. The results show that HNAC crystallizes in the monoclinic space group *P*2_1_/*c*, with two mol­ecules in the asymmetric unit, stabilized by an extensive network of intra- and inter­molecular hy­dro­gen bonds. This well-organized hy­dro­gen-bonding framework underpins the robust crystal packing and thermal stability of the com­pound, as further corroborated by thermal analysis and powder X-ray diffraction. Complementary IR spectroscopy confirmed the presence of key functional groups and highlighted the impact of hy­dro­gen bonding on vibrational modes.

Placing these findings in the broader context of lipid-based permeation enhancers, HNAC retains structural features typical of other medium-chain fatty acids, such as extended aliphatic chain conformations, while also introducing an additional aromatic amide fragment. This distinguishes HNAC from other medium-chain fatty acids which act as permeation enhancers, like hepta­noic acid and suberic acid. Crystallographically, this study shows the difference between simpler and more com­plex lipid-based permeation enhancers. The insights into the mol­ecular conformation and solid-state inter­actions of HNAC could enhance our understanding of its physicochemical properties, providing a valuable structural foundation for the development and optimization of SNAC-based drug delivery systems and related permeation enhancers.

## Supplementary Material

Crystal structure: contains datablock(s) I, global. DOI: 10.1107/S2053229625008691/vp3046sup1.cif

Structure factors: contains datablock(s) I. DOI: 10.1107/S2053229625008691/vp3046Isup3.hkl

Supporting information file. DOI: 10.1107/S2053229625008691/vp3046Isup3.cml

PXRD patterns. DOI: 10.1107/S2053229625008691/vp3046sup4.pdf

CCDC reference: 2451829

## Figures and Tables

**Table 1:** Experimental details

Crystal data	
Chemical formula	C_15_H_21_NO_4_
*M*_r_	279.33
Crystal system, space group	Monoclinic, *P*2_1_/*c*
Temperature (K)	150
*a*, *b*, *c* (Å)	10.1779 (2), 23.7511 (6), 11.8738 (3)
β (°)	101.124 (2)
*V* (Å^3^)	2816.40 (12)
*Z*	8
Radiation type	Cu *K*α
μ (mm^−1^)	0.78
Crystal size (mm)	0.19 × 0.04 × 0.02

Data collection	
Diffractometer	Rigaku OD XtaLAB Synergy-S HyPix-Arc 100
Absorption correction	Analytical [*CrysAlis PRO* (Rigaku OD, 2024 ▸) based on expressions derived by Clark & Reid (1995 ▸)]
*T*_min_, *T*_max_	0.922, 0.989
No. of measured, independent and observed [*I* > 2σ(*I*)] reflections	21630, 5561, 4555
*R*_int_	0.036
(sin θ/λ)_max_ (Å^−1^)	0.633

Refinement	
*R*[*F*^2^ > 2σ(*F*^2^)], *wR*(*F*^2^), *S*	0.046, 0.131, 1.06
No. of reflections	5561
No. of parameters	379
H-atom treatment	H atoms treated by a mixture of independent and constrained refinement
Δρ_max_, Δρ_min_ (e Å^−3^)	0.31, −0.18

Computer programs: *CrysAlis PRO* (Rigaku OD, 2024 ▸), *SHELXT2018* (Sheldrick, 2015 ▸), *SHELXL* (Sheldrick, 2008 ▸) and *OLEX2* (Dolomanov *et al.*, 2009 ▸).

**Table 2:** Hydrogen-bond geometry (Å, °)

*D*—H⋯*A*	*D*—H	H⋯*A*	*D*⋯*A*	*D*—H⋯*A*
O1—H1⋯O2	0.89 (2)	1.76 (2)	2.5737 (16)	151 (2)
O4—H4⋯O2^i^	0.93 (2)	1.73 (2)	2.6637 (15)	177 (2)
O5—H5*A*⋯O6	0.87 (2)	1.76 (2)	2.5660 (15)	153.1 (19)
O8—H8⋯O6^ii^	0.94 (2)	1.71 (2)	2.6479 (15)	176.4 (19)
N1—H1*A*⋯O7	0.887 (19)	2.15 (2)	3.0220 (17)	169.3 (17)
N2—H2⋯O3	0.905 (19)	2.078 (19)	2.9708 (17)	168.6 (16)

Symmetry codes: (i) 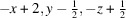; (ii) 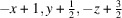.
