# Supplementary material for: Crystallographic and physicochemical characterization of salcaprozoic acid: a structural basis for SNAC-enabled drug delivery systems
Source: Acta Crystallogr C Struct Chem. 2025 Oct 6;81(Pt 11):607–13. doi: 10.1107/S2053229625008691 (PMC12587319; doi:10.1107/S2053229625008691)
Supplement: Supplementary file 4 [file c-81-00607-sup4.pdf]

STRUCTURAL CHEMISTRY

Volume 81 (2025)

Supporting information for article:

Crystallographic and physicochemical characterization of salcaprozoic acid: a structural basis for SNAC-enabled drug delivery systems

Parag Roy, Paul G. Waddell, Rajdeep Dey and Oisín N. Kavanagh

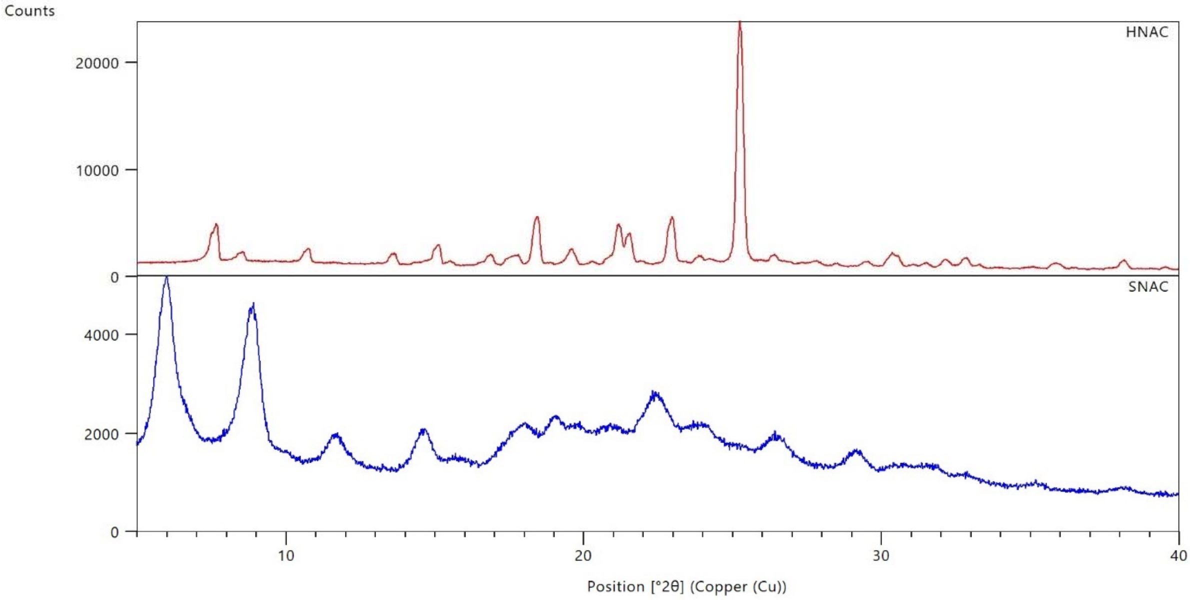

Figure S1. PXRD patterns of HNAC and SNAC.
